# The Reliability of the Arabic Version of the Dyspnea Index Questionnaire for Upper Airway-Related Dyspnea

**DOI:** 10.7759/cureus.29656

**Published:** 2022-09-27

**Authors:** Abdulaziz Alrabiah, Bshair Aldriweesh, Nasser Almujaiwel, Mohammad I Alasqah, Shatha A Alduraywish, Ahmad Alammar

**Affiliations:** 1 Department of Otorhinolaryngology, Prince Sultan Military Medical City, Riyadh, SAU; 2 Department of Otorhinolaryngology, King Fahad Specialist Hospital, Dammam, SAU; 3 Department of Otorhinolaryngology, College of Medicine, King Saud University Medical City, King Saud University, Riyadh, SAU; 4 Department of Otolaryngology, College of Medicine, King Saud University, Riyadh, SAU; 5 Department of Family and Community Medicine, College of Medicine, King Saud University, Riyadh, SAU

**Keywords:** translation and validation, validation, upper airway obstruction, quality of life, laryngotracheal stenosis, patient-reported outcome measures, dyspnea index

## Abstract

Objectives

This study aimed to translate the Dyspnea Index (DI) questionnaire into the Arabic language and determine whether this version is valid and reliable for Arabic-speaking patients with upper airway-related dyspnea.

Methods

A cross-sectional study was conducted at the King Saud University Medical City otolaryngology clinics in Riyadh, Saudi Arabia. The DI questionnaire was translated into Arabic and then back-translated into the English language. Inclusion criteria were preoperative patients presenting to the otolaryngology clinic with upper airway-related dyspnea between November and December 2020. The results of internal consistency and factor analysis among the items were compared to the original DI development results to assess the reliability of the questionnaire.

Results

Among a total of 57 recruited patients, 50 questionnaires were completed with an 88% response rate. The mean age of the included patients was 38 ±14 years. Women constituted 58% of the patients. The most common diagnosis was subglottic stenosis (72%). Principle component extraction in factor analysis revealed a single underlying factor for all the questions. Factor loading ranged from 0.69 to 0.85. Reliability statistics showed a high value of internal consistency among the items. The mean inter-item correlation was 0.58.

Conclusion

Based on our findings, the Arabic version of the DI questionnaire is a reliable instrument for evaluating upper airway dyspnea.

## Introduction

Dyspnea has several different definitions; one known definition is the subjective somatopsychic feeling of breathlessness at rest or with limited effort, causing considerable distress to people and their caregivers across the community [1]. The prevalence of adults with dyspnea that restricts daily life activities on a long-term basis was found to be 8.9% in a population-based study in South Australia [2]. The causes of breathlessness include a combination of pathologies encompassing chronic lung and heart diseases, malignancies, chronic infections, anemia, progressive muscle weakness, obesity, and anxiety [3]. Upper airway-related dyspnea is characterized by a group of disabling diseases and conditions that are underrecognized and vary in severity. They are categorized into structural, functional, and neurological characteristics. In the structural category, such as laryngotracheal stenosis and vocal fold ankylosis, the patients present with breathing difficulties on exertion and even at rest in severe cases [4,5]. In the functional category, e.g., paradoxical vocal fold motion (PVFM), the symptoms occur upon exposure to a triggering factor [5,6]. Lastly, in the neurological category, the symptoms can occur continuously, such as bilateral vocal fold paralysis, or periodically, like laryngeal dystonia [5,7]. The management of upper airway-related dyspnea varies depending on the underlying pathology; it ranges from urgent interventions for patients with acute respiratory distress to supportive non-surgical therapy in an outpatient setting [4]. Accurately differentiating between upper and lower airway pathologies and their presentations is vital for guiding patient management [8]. Diseases that affect the upper airway may have common symptoms apart from dyspnea, such as hoarseness, sleep disturbance, choking, dry cough, the sensation of a lump in the throat, and noisy breathing. Certainly, dyspnea is the predominant symptom [5,9].

There are several methods mentioned in the literature for the evaluation of dyspnea. Psycho-physical testing is defined as the estimation of awareness of changes in breathing in response to externally added loads [10,11]. Although this approach has led to a greater understanding of dyspnea, its application in clinics is limited by several factors, including technical aspects and time requirements. Physiological testing, in the form of pulmonary function tests, can be helpful to assess the severity of the obstructive condition [12,13]. However, understanding the full impact of the condition on patients’ lives and their satisfaction is equally or somewhat more vital in management decision-making [4]. Moreover, repeating these physical tests may not be convenient for patients. Therefore, there is a need for a reliable assessment tool to measure and quantify the severity of dyspnea. Many self-report questionnaires have been developed in the literature to assess the severity of dyspnea; some of these questionnaires target dyspnea in general [14,15] or specifically target lower respiratory tract-related dyspnea, such as the chronic obstructive pulmonary disease assessment test questionnaire [16].

The Dyspnea Index (DI) questionnaire is a valid and reliable 10-item patient-centered instrument developed by Gartner-Schmidt et al. that can be used for both the initial and follow-up assessments of the severity of dyspnea. The questionnaire was originally developed with 41 items that were subsequently reduced by principal component analysis to the current 10 items. It is graded on a 5-point Likert scale that guides in evaluating the treatment outcomes and drives evidence-based clinical decisions. The questionnaire was found to be an effective and convenient assessment tool that can be administered in an outpatient setting [6]. The only dyspnea assessment Arabic questionnaires found in the literature target dyspnea in chronic obstructive pulmonary disease patients [17,18]. Moreover, this questionnaire targeting upper airway diseases, in particular, was translated and validated in Swedish [19]. Yet, it has not been translated into Arabic. The availability of a validated Arabic questionnaire could aid in the management of Arabic-speaking patients suffering from upper airway-related dyspnea. It will also contribute to future research in the region and further improve the knowledge related to patient-specific care and outcomes for Arabic-speaking patients. This study aims to translate the DI 10-item questionnaire into Arabic and establish its reliability and validity for upper airway-related dyspnea.

## Materials and methods

Translation and cross-cultural adaptation process

Using the forward and backward translation method, The DI questionnaire was translated separately into Arabic version by two fluent bilingual experts in the otolaryngology and airway surgery fields (the first and second authors, both native Arabic and fluent English speakers). Then an expert panel (one consultant in otolaryngology and airway surgery field, the last author) reviewed it before it was back-translated into English by a bilingual licensed English translator with a medical background who had not been exposed to the original questionnaire. Later, interviews with native Arabic speakers of different backgrounds were carried out to omit any possible unclear or vague expressions in the questionnaire. For reconciliation, discrepancies and issues in the copies of each stage during the process were discussed thoroughly and resolved before proceeding to the second. The discussion took into consideration the original DI questionnaire and the cultural and language differences before arriving at the most appropriate and suitable wordings for the translation of the questionnaire.

Survey

The questionnaire consisted of informed consent at the outset, followed by basic demographic information, including name, gender, and date of birth. Moreover, it included 10 questions related to dyspnea, including its location, perceived sense of worsening, weather, stress, or exercise aggravation, presence of stridor, or straining, and its effect on stress or social life. All were rated according to a 5-point Likert scale. On this scale, 0 stands for ‘never,’ and 4 stands for ‘always’. Questions were selected intentionally to reflect the latent variables they were designed to measure. Other variables such as comorbidities, previous surgeries, and diagnosis of upper airway pathology were obtained from their medical files.

Data collection and participants

This cross-sectional study was conducted at the King Saud University Medical City otolaryngology clinics in Riyadh, Saudi Arabia. Preoperative patients presenting to otolaryngology clinics with upper airway-related dyspnea were involved in assessing the translated version of the questionnaire. The inclusion criteria were preoperative patients presenting to otolaryngology clinics with upper airway-related dyspnea, having a minimum age of 18 years, irrespective of their gender. Those patients were selected by a simple random sampling technique from the list of scheduled future surgeries in the institute. Postoperative patients and patients aged less than 18 years were excluded. The original sample size consisted of 57 patients. Data were collected over a two-month period between November and December 2020; the online survey questionnaire was sent on the preferred digital medium of the included participants.

Ethical considerations

The approval of this study was obtained from the Institutional Review Board of the College of Medicine at King Saud University, Riyadh, Kingdom of Saudi Arabia, with the assigned project number E-21-5704. Permission was obtained from the developer for the use and translation of the questionnaire. Before the commencement of the survey, patients were assured of their anonymity, and their consent was obtained. No incentives or rewards of any kind were offered to the participants; they were informed of the purpose of the study, and they had the right to withdraw at any time with no obligation toward the study team. The authors of this study have no conflict of interest.

Statistical analysis

SPSS Statistics version 24.0 (IBM Corp., Armonk, NY) was used for data analysis. Descriptive statistics (means, standard deviations, and frequencies) were used to report demographic data and scale items. The internal consistency among the items was evaluated by Cronbach's alpha (a value of 0.7 or higher was considered significant). Factor analysis was performed to allocate items to the appropriate underlying factor (factor loading of more than 0.3 was considered significant). A p-value of 0.05 or less was considered statistically significant.

## Results

Demographic and baseline data

Out of 57 recruited patients, 50 patients agreed to participate in this study (response rate: 88%). In terms of gender, there were 29 females (58%). The mean age was 38.14 ±14 years (range: 18-72 years), and the age was normally distributed. The most common diagnosis among the participants was subglottic stenosis (72%), followed by tracheal stenosis (12%), vocal cord paralysis (8%), and combined subglottic and tracheal stenosis (2%) (Table 1).

**Table 1:** Causes of upper airway-related dyspnea in the study participants

Diagnosis	Frequency (n = 50)	Percentage
Subglottic stenosis	36	72.0
Tracheal stenosis	6	12.0
Combined subglottic and tracheal stenosis	1	2.0
Vocal fold paralysis	4	8.0
Others	2	4.0
Missing	1	2.0
Total	50	100.0

The Dyspnea Index questionnaire

Analysis of patients’ responses to the questionnaire revealed that the responses ranged from 0 to 4 on all questions (mean: 1.75 ±0.29) (Table 2). Principle component extraction in factor analysis revealed a single underlying factor for all the questions. Loading factors’ coefficient (communalities) ranged from 0.69 to 0.85 (Table 3). Reliability statistics showed a high value of internal consistency among the items (Cronbach’s alpha = 0.93). The mean inter-item correlation was 0.58, as shown in Table 4.

**Table 2:** Patient response to the questionnaire

Questions	N	Mean ±standard deviation
1. I have trouble getting air in	50	1 ±1.12
2. I feel tightness in my throat when I am having a breathing problem	50	2 ±1.47
3. It takes more effort to breathe than it used to	50	2 ±1.42
4. Changes in weather affect my breathing problem	50	2 ±1.51
5. My breathing gets worse with stress	50	2 ±1.44
6. I make sound/noise breathing in	50	2 ±1.50
7. I have to strain to breathe	50	2 ±1.32
8. My shortness of breath gets worse with exercise or physical activity	50	2 ±1.60
9. My breathing problem makes me feel stressed	50	2 ±1.32
10. My breathing problem causes me to restrict my personal and social life	50	2 ±1.50

**Table 3:** Principal component extraction ^a^1 component extracted Extraction method: principal component analysis

Component matrix^a^	
Item	Component
Item 1	0.80
Item 2	0.85
Item 3	0.82
Item 4	0.77
Item 5	0.80
Item 6	0.76
Item 7	0.82
Item 8	0.76
Item 9	0.77
Item 10	0.69

**Table 4:** Inter-item correlation matrix

	Item 1	Item 2	Item 3	Item 4	Item 5	Item 6	Item 7	Item 8	Item 9	Item 10
Item 1	1.00	0.65	0.67	0.76	0.71	0.59	0.55	0.53	0.51	0.32
Item 2	0.65	1.00	0.64	0.61	0.64	0.65	0.73	0.64	0.58	0.49
Item 3	0.67	0.64	1.00	0.53	0.62	0.65	0.60	0.55	0.64	0.51
Item 4	0.76	0.61	0.53	1.00	0.71	0.40	0.55	0.42	0.62	0.46
Item 5	0.71	0.64	0.62	0.71	1.00	0.50	0.57	0.46	0.63	0.40
Item 6	0.59	0.65	0.65	0.40	0.50	1.00	0.62	0.73	0.38	0.43
Item 7	0.55	0.73	0.60	0.55	0.57	0.62	1.00	0.62	0.58	0.61
Item 8	0.53	0.64	0.55	0.42	0.46	0.73	0.62	1.00	0.49	0.60
Item 9	0.51	0.58	0.64	0.62	0.63	0.38	0.58	0.49	1.00	0.67
Item 10	0.32	0.49	0.51	0.46	0.40	0.43	0.61	0.60	0.67	1.00

## Discussion

There are several patient-reported outcome (PRO) questionnaires designed for the assessment and monitoring of patients with upper airway obstruction [15,20]. However, the DI is the only tool that was developed with the input of participants with different and various upper airway pathologies [6,21]. The primary objective of this study was to translate the DI questionnaire into the Arabic language and to determine whether the Arabic version of the DI is a reliable source to be administered in otolaryngology clinics. The results of this study showed that the Arabic version of DI shows good psychometric properties and could be implemented in otolaryngology clinics to evaluate and follow up on Arabic-speaking patients with dyspnea related to upper airway pathology.

A study comparing the role of spirometry and DI in the management of subglottic stenosis showed that DI has a good fit but does not have a solid correlation with pulmonary function test measurements. While DI remarkably improves the treatment of subglottic stenosis, it is not sensitive or sufficiently specific to be used alone to make clinical decisions in these patients [22]. Although its routine utilization in clinical practice has not yet been established, the questionnaire is expected to be helpful as a straightforward tool to enhance our understanding of how patients perceive the outcomes of various care strategies. This emphasizes the necessity for additional studies centered on the significance and perhaps other applications and uses of the DI.

To compare our results with the original study [6], a principal component extraction in factor analysis revealed a single underlying factor for all the questions. This is similar to the original study in which a single factor was found underlying the items. The factor loading ranged from 0.69 to 0.85 in our study, while factor loadings of 0.70 or greater for all the 10 items were found in the English version of the questionnaire. Reliability statistics showed a high value of internal consistency among the items (Cronbach’s alpha = 0.93). Comparably, in their study analysis, the internal consistency was also high (Cronbach’s alpha = 0.91). In comparison to different studies done in DI, there is a Swedish version of DI with Cronbach's alpha of 0.85 in which they conclude that their version is reliable [19]. In a study testing DI in adolescents aged 12-18 years with exercise-induced PVFM, the authors determined their Cronbach's alpha to be 0.80 and also reliable. The mean inter-item correlation in our study was 0.58; in the PVFM study mentioned earlier, the intraclass correlation coefficient was calculated to be 0.8, which implies substantial reliability. The results support that the Arabic DI accurately assesses upper airway-related dyspnea, similar to the findings in the literature [21].

This study is limited by its cross-sectional design, which was carried out using an online self-administered survey; the choice was made according to the contact precaution guidelines at the time of the data collection. However, this allowed us to reach a much broader variety of participants from different districts and cities with diverse sociodemographic backgrounds and linguistic types. Additionally, the vast majority of the sample was diagnosed with subglottic stenosis, which was due to the rarity of this presenting complaint; this might have a biased effect on the results considering the need for a more variant pathology in the sample. In addition to the absence of repeatability testing, the study's sample size is another drawback, although the number of participants enrolled is comparable to that of the questionnaire's original validation. Nevertheless, the collected sample size was enough to show significant results and is considered reliable. Also, the Arabic DI could not be compared to another same-language questionnaire due to the absence of a validated Arabic-language PRO instrument exclusively targeting patients with upper airway dyspnea, which reinforces the value and necessity of this validation.

## Conclusions

This is by far the first Arabic questionnaire targeting upper airway dyspnea. The Arabic version of the DI is a reliable source to evaluate upper airway dyspnea. Our results will hopefully encourage researchers and clinicians to use the Arabic DI in future studies to further understand the importance and possibly the additional broader uses of the DI. Furthermore, designing or translating other questionnaires to cover all types of dyspnea, such as for those suffering from both upper and lower airway dyspnea, will enhance healthcare and save resources.

## Figures and Tables

**Figure 1:**
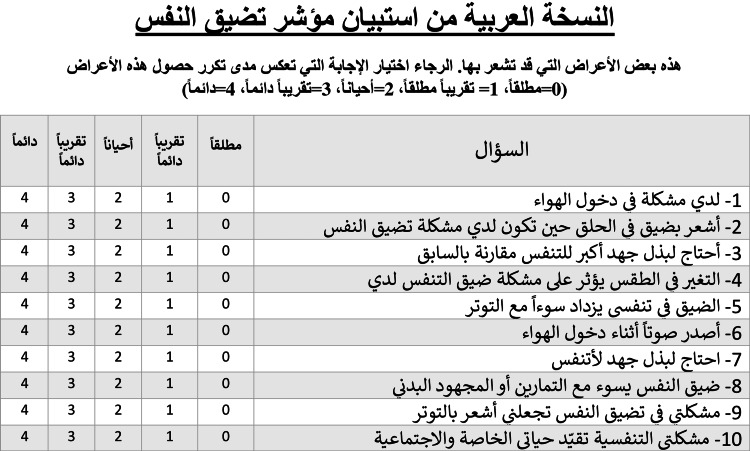
The Arabic version of the Dyspnea Index

**Figure 2:**
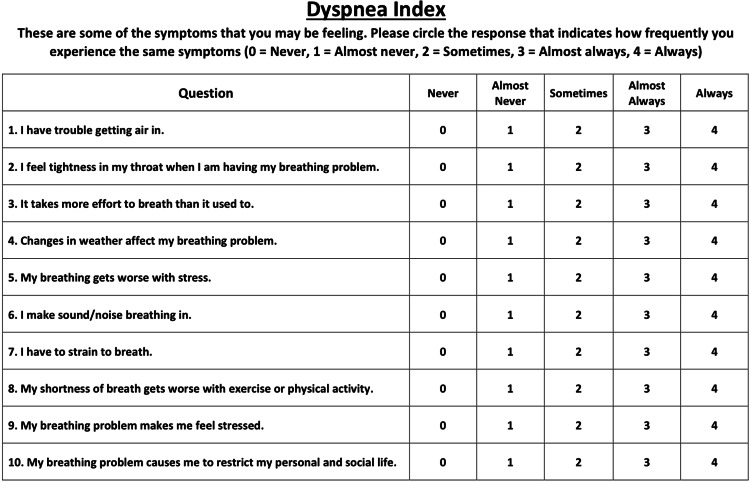
The Dyspnea Index Permission was obtained from the developer [6]
